# Characterization of the Landscape of Joint MD/MBA Programs in the US, 2002 to 2022

**DOI:** 10.1001/jamanetworkopen.2023.21268

**Published:** 2023-06-30

**Authors:** Folawiyo Laditi, Wilton Sun, Howard P. Forman

**Affiliations:** 1 Yale University School of Medicine, New Haven Connecticut; 2 Yale School of Management, New Haven, Connecticut; 3 Department of Radiology, Yale School of Medicine, New Haven, Connecticut; 4 Yale School of Public Health, New Haven, Connecticut

## Abstract

This cross-sectional study characterizes the landscape of joint MD/MBA programs in the US from 2002 to 2022.

## Introduction

For medical students interested in health care leadership roles, joint doctor of medicine and master of business administration (MD/MBA) programs are a popular option. How schools administer these dual-degree programs varies greatly, raising questions about curriculum integration, educational value, and outcomes.^1,2^

In this 20-year follow-up to a study by Larson et al^3^ on MD/MBA programs in the US as of 2002, web research was conducted to characterize the current landscape and illustrate consistent features.

## Methods

From May to June 2022, we compiled a comprehensive list of MD/MBA programs across the US. We collected information on medical schools and their associated business schools, using basic internet searches, email follow-up, and online resources (eMethods in Supplement 1). This cross-sectional study did not involve human participants and was deemed exempt from review and informed consent by Yale University under the Common Rule. The study followed the STROBE reporting guideline. Statistical analyses were conducted with R, version 3.5.3, with significance at .05 using 2-tailed *P* values. Data analyses were performed on September 16, 2022.

## Results

In 2002, 33 of 125 US medical schools (26.4%) offered MD/MBA programs.^3^ In 2022, we identified 92 MD/MBA programs among 151 US medical schools (60.9%), including 60 programs not identified in 2002 (Table 1). A total of 90 of 91 programs (98.0%) were completable within 5 years, and 70 of 77 (90.9%) had different MBA credit requirements when completed in the dual-degree program vs the stand-alone MBA. Only 18 of 70 programs (25.7%) required business internships during the dual degree, and 28 of 83 (33.7%) required an admissions test for the business school portion.

**Table 1.** Characteristics of MD/MBA Programs in the US Established Before or During 2002 vs After 2002^a^

Characteristic	MD/MBA programs			*P* value
	Established in 2002 or earlier (n = 32)	Established after 2002 (n = 60)	Total (N = 92)	
Medical school operation or ownership type				
Private	17 (53.1)	18 (30.0)	35 (38.0)	.03
Public	15 (46.9)	42 (70.0)	57 (62.0)	
MD and MBA offered at the same institution?				
No	3 (9.4)	11 (18.3)	14 (15.2)	.26
Yes	29 (90.6)	49 (81.7)	78 (84.8)	
Website mentions MBA courses available partially or exclusively online?				
Data not available^b^	1	0	1	.13
No	29 (93.5)	49 (81.7)	78 (85.7)	
Yes	2 (6.5)	11 (18.3)	13 (14.3)	
MBA credits differ if completing MBA as part of dual degree vs MBA alone				
Data not available	7	8	15	.05
No	0 (0.0)	7 (13.5)	7 (9.1)	
Yes	25 (100.0)	45 (86.5)	70 (90.9)	
Program length, y				
Data not available	1	0	1	.66
4	2 (6.5)	7 (11.7)	9 (9.9)	
4-5	1 (3.2)	5 (8.3)	6 (6.6)	
5	27 (87.1)	46 (76.7)	73 (80.2)	
5-6	1 (3.2)	1 (1.7)	2 (2.2)	
≤7	0 (0.0)	1 (1.7)	1 (1.1)	
Unique courses for MD/MBAs?				
Data not available	3	13	16	.79
No	26 (89.7)	43 (91.5)	69 (90.8)	
Yes	3 (10.3)	4 (8.5)	7 (9.2)	
Can apply to the MBA before medical school matriculation?				
Data not available	2	8	10	.51
No	18 (60.0)	35 (67.3)	53 (64.6)	
Yes	12 (40.0)	17 (32.7)	29 (35.4)	
Students must apply separately to MD and MBA programs				
Data not available	1	4	5	.68
No	3 (9.7)	4 (7.1)	7 (8.0)	
Yes	28 (90.3)	52 (92.9)	80 (92.0)	
MBA portion requires GMAT or GRE for admission?				
Data not available	3	6	9	.01
No	14 (48.3)	41 (75.9)	55 (66.3)	
Yes	15 (51.7)	13 (24.1)	28 (33.7)	
MBA has preadmissions employment requirement?				
Data not available	2	2	4	.47
No	30 (100.0)	57 (98.3)	87 (98.9)	
Yes	0 (0.0)	1 (1.7)	1 (1.1)	
MBA credits count toward medical school requirements				
Data not available	18	35	53	.66
No	2 (14.3)	5 (20.0)	7 (17.9)	
Yes	12 (85.7)	20 (80.0)	32 (82.1)	
Medical school credits count toward MBA requirements				
Data not available	20	22	42	.65
No	1 (8.3)	5 (13.2)	6 (12.0)	
Yes	11 (91.7)	33 (86.8)	44 (88.0)	
Summer internship is MBA graduation requirement				
Data not available	8	14	22	<.001
No	12 (50.0)	40 (87.0)	52 (74.3)	
Yes	12 (50.0)	6 (13.0)	18 (25.7)	
Is MBA completely separate? (time to completion is same as MBA only)				
Data not available	3	5	8	.34
No	28 (96.6)	50 (90.9)	78 (92.9)	
Yes	1 (3.4)	5 (9.1)	6 (7.1)	
USMLE examinations must be partially or fully completed before MBA matriculation				
Data not available	10	24	34	.36
No	14 (63.6)	27 (75.0)	41 (70.7)	
Yes	8 (36.4)	9 (25.0)	17 (29.3)	
Fee waiver for MBA admissions given to MD students				
Data not available	23	53	76	.34
No	6 (66.7)	3 (42.9)	9 (56.2)	
Yes	3 (33.3)	4 (57.1)	7 (43.8)	

Abbreviations: GMAT, Graduate Management Admission Test; GRE, Graduate Record Examination; MBA, master of business administration; MD, doctor of medicine; USMLE, US Medical Licensing Examination.

^a^ Unless indicated otherwise, values are presented as the No. (%) of programs.

^b^ Values are the No. of programs.

We compared programs that existed at the time of the 2002 study^3^ (old programs) with those newly identified since (new programs). Table 1 reveals 3 significant differences between old vs new programs: a higher proportion of old programs required summer internships as part of the MBA (12 [50.0%] vs 6 [13.0%]; *P* < .001), were private institutions (17 [53.1%] vs 18 [30.0%]; *P* = .03), and required an admissions test for the MBA studies (15 [51.7%] vs 13 [24.1%]; *P* = .01).

We categorized programs based on their curriculum organization as defined in our 2002 study and added a new category for the 20 programs that offered at least 2 of the original models (Table 2). Of the 87 available curricula, 39 (44.8%) followed model 1.

**Table 2.** Curriculum Sequence Models Followed by MD/MBA Programs in the US, 2022^a^

Model	No. of MD/MBA programs, % (n = 87)	Year in program				
		1	2	3	4	5
1	39 (44.8)	Basic science	Basic science	Clinical rotations	Management	Clinical rotations and management
2	10 (11.5)	Basic science	Basic science	Management	Clinical rotations	Clinical rotations and management
3	5 (5.6)	Management	Basic science	Basic science	Clinical rotations	Clinical rotations
4	1 (1.2)	Basic science	Management	Basic science	Clinical rotations	Clinical rotations
5	5 (5.8)	Basic science and management	Basic science and management	Clinical rotations	Clinical rotations and management	NA
6	20 (23.0)	At least 2 of sequence models 1-5 offered	At least 2 of sequence models 1-5 offered	At least 2 of sequence models 1-5 offered	At least 2 of sequence models 1-5 offered	At least 2 of sequence models 1-5 offered
Other^b^	7 (7.9)	NA	NA	NA	NA	NA

Abbreviations: MBA, master of business administration; MD, doctor of medicine; NA, not applicable.

^a^ We categorized the available curricula of 87 MD/MBA programs in the US based on the sequence of completion of medical school and business school requirements as defined in Larson et al.^3^ We added a new category for programs that offered at least 2 of the first 5 original sequences for students to choose from during their studies. For the 5 programs that are not listed, their curriculum sequences were not available online.

^b^ Within this category, 3 programs individualized the timing of MBA coursework to student preferences and therefore did not follow a defined sequence, 3 programs had students split their final 2 years between the medical and business schools, and 1 program had students complete MBA coursework after the completion of their fourth year of medical school.

## Discussion

We observed that the percentage of schools offering MD/MBA programs increased from 26.4% in 2002 to 60.9% in 2022, offering more students such education.^3,4^ Recent data suggest that 0.8% of medical students graduate with an MD/MBA, equating to roughly 160 students compared with 61 in 2002.^3,5^ Notably, the MD/MBA programs typically took 5 or fewer years to complete and required fewer business school credits compared with stand-alone MBAs. Many programs also offered online options, reflecting their focus on meeting the unique educational needs of medical students while providing flexibility. Some programs have multiple sequence models available, demonstrating further adaptability in curriculum design.

A lower proportion of new programs required a (business) internship, offering more flexibility. Compared with older programs, the increased availability of MBA programs in public medical schools should make these programs more accessible to more students.^6^

The primary limitation of our study is that we relied predominantly on web-based research for data gathering. Regrettably, the paucity of available information restricted our capacity to report on all desired features comprehensively. Although some communication was made with programs to authenticate data, it remains uncertain whether the captured information precisely represents program functions or if any program lacked registered students.

In this cross-sectional study, we identified 92 MD/MBA programs across the US, with the majority being completable in 5 years. These programs exhibit substantial variation, with flexibility in program requirements emerging as an important theme. Overall, like the executive MBA, the MD/MBA dual-degree program remains a popular option for medical and business schools seeking to train leaders in health care.

## Notes

Supplement 1.
eMethods

eReferences

Supplement 2.
Data Sharing Statement
