# Supplementary material for: Characterization of the Landscape of Joint MD/MBA Programs in the US, 2002 to 2022
Source: JAMA Netw Open. 2023 Jun 30;6(6):e2321268. doi: 10.1001/jamanetworkopen.2023.21268 (PMC10314300; doi:10.1001/jamanetworkopen.2023.21268)
Supplement: Supplement 1. — eMethods eReferences [file jamanetwopen-e2321268-s001.pdf]

Supplemental Online Content

Laditi F, Sun W, Forman HP. Characterization of the landscape of joint MD/MBA programs in the US, 2002 to 2022. *JAMA Netw Open*. 2023;6(6):e2321268. doi:10.1001/jamanetworkopen.2023.21268

eMethods
eReferences

This supplemental material has been provided by the authors to give readers additional information about their work.

© 2023 Laditi F et al. *JAMA Network Open.*

eMethods

Data Collection

We specifically defined an MD/MBA program as one where there is a formalized partnership between the medical school and a business school (not necessarily within the same university). We used a variety of resources including basic internet searches, the AAMC MSAR tool, and the Association of MD/MBA programs website to collect information on medical school and business school characteristics.[1,2] We also referred to past studies on MD/MBA programs.[3,4] For programs that were mentioned in one resource but a current website could not be found, we contacted deans of student affairs to confirm or deny their existence. We obtained information on the state, AAMC region, and ownership/control status (i.e., private or public) of each MD/MBA program from the AAMC Medical School Organizational Characteristics Database, and used this resource to determine that there are currently 151 US MD-granting programs for the calculation of prevalence of MD/MBA programs amongst medical schools.[5] Additional information about the MD/MBA programs was obtained from their medical school and/or business school pages specific to these joint degrees. A second researcher double-checked this work by reviewing the program websites independently. To determine the US Census region, we compared the medical school state information to US census regions (Supplement Table 1).

The Wake Forest School of Medicine MD/MBA program is no longer in operation due to the MBA program's shift from full-time to online and part-time delivery. We confirmed the transition of the Wake Forest MD/MBA program to an online degree format through email. Although the UC Davis program is mentioned on the medical and business school pages, and the association of MD/MBA programs website, no specific program page could be found, and email

© 2023 Laditi F et al. *JAMA Network Open.*

requests went unanswered. However, given the number of online mentions from official sources, we have included it in our study.

This study was exempt from institutional human investigation committee review. This study adhered to the Strengthening the Reporting of Observational Studies in Epidemiology (STROBE) reporting guideline for observational studies.[6]

Statistical Analyses

Data analysis was conducted using R 3.5 software.[7] Basic descriptive statistics, including mean, median, and standard deviation, were run on the data set to provide an overview of the sample. Student's t-test and ANOVA functions were used to analyze and determine if any significant differences existed between the older and newer program groups.[8] Additionally, we used the chi-squared test to analyze and determine if any significant associations existed between the program groups and geographic areas or MD/MBA sequence models. This report follows STROBE guidelines for observational studies.

eReferences

1. Association of American Medical Colleges. Medical School Admission Requirements (MSAR) for Applicants. AAMC Students & Residents. Published 2022. Accessed June 8, 2022. https://students-residents.aamc.org/medical-school-admission-requirements/medical-school-admission-requirements-msar-applicants

2. Association of MD/MBA programs. MD/MBA Programs. Association of MD/MBA Programs Healing Healthcare. Published 2019. Accessed May 15, 2022. http://www.mdmbaprograms.org/md-mba-programs/

3. Larson DB, Chandler M, Forman HP. MD/MBA programs in the United States: evidence of a change in health care leadership. *Acad Med*. 2003;78(3):335-341.

4. Keogh TJ, Martin WM. *The Convergence of Business and Medicine: A Study of MD/MBA Programs in the United States*. Education Resources Information Center; 2011.

© 2023 Laditi F et al. *JAMA Network Open.*

5. Association of American Medical Colleges. Organizational Characteristics Database. *Teaching Hospital Affiliates*. Published online June 10, 2022. Accessed August 28, 2022. https://www.aamc.org/data-reports/faculty-institutions/report/organizational-characteristics-database-ocd

6. von Elm E, Altman DG, Egger M, et al. The Strengthening the Reporting of Observational Studies in Epidemiology (STROBE) Statement: guidelines for reporting observational studies. *Int J Surg*. 2014;12(12):1495-1499.

7. R Foundation for Statistical Computing, Vienna, Austria. *R Core Team (2022). R: A Language and Environment for Statistical Computing*.; 2021. https://www.R-project.org/

8. Heinzen E, Sinnwell J, Atkinson E, et al. arsenal: An Arsenal of “R” Functions for Large-Scale Statistical Summaries. R package version. 2019;3.

© 2023 Laditi F et al. *JAMA Network Open.*
