# Supplementary material for: Characterization of the Landscape of Joint MD/MBA Programs in the US, 2002 to 2022
Source: JAMA Netw Open. 2023 Jun 30;6(6):e2321268. doi: 10.1001/jamanetworkopen.2023.21268 (PMC10314300; doi:10.1001/jamanetworkopen.2023.21268)
Supplement: Supplement 2. — Data Sharing Statement [file jamanetwopen-e2321268-s002.pdf]

Data Sharing Statement

Laditi. Characterization of the Landscape of Joint MD/MBA Programs in the US, 2002 to 2022. *JAMA Netw Open*. Published June 30, 2023. doi:10.1001/jamanetworkopen.2023.21268

Data
Data available: Yes
Data types: Data (not involving human participants)
How to access data: Folaladiti6@gmail.com
When available: With publication

Supporting Documents
Document types: None

Additional Information
Who can access the data: anyone requesting the data
Types of analyses: for any purpose
Mechanisms of data availability: no restrictions
